# Improving Image Quality and Decreasing SAR With High Dielectric Constant Pads in 3 T Fetal MRI

**DOI:** 10.1002/jmri.29677

**Published:** 2025-01-21

**Authors:** Zhengyang Zhu, Xunwen Xue, Tang Tang, Chao Luo, Ye Li, Jing Chen, Biyun Xu, Zengping Lin, Xin Zhang, Zhengge Wang, Jun Chen, Jiaming Lu, Wen Zhang, Xin Li, Qian Chen, Zhuoru Jiang, Junxia Wang, Qing Hu, Sven Haller, Ming Li, Chenchen Yan, Bing Zhang

**Affiliations:** ^1^ Department of Radiology, Nanjing Drum Tower Hospital, Affiliated Hospital of Medical School Nanjing University Nanjing Jiangsu China; ^2^ Medical Imaging Center, Affiliated Drum Tower Hospital Medical School of Nanjing University Nanjing Jiangsu China; ^3^ Nanjing University Institute of Medical Imaging and Artificial Intelligence Nanjing University Nanjing Jiangsu China; ^4^ Lauterbur Research Center for Biomedical Imaging, Shenzhen Institutes of Advanced Technology Chinese Academy of Sciences Shenzhen China; ^5^ Shenzhen Key Laboratory for MRI Shenzhen China; ^6^ Medical Statistics and Analysis Center, Nanjing Drum Tower Hospital, Affiliated Hospital of Medical School Nanjing University Nanjing Jiangsu China; ^7^ Central Research Institute United Imaging Healthcare Group Co., Ltd Shanghai China; ^8^ CIMC Geneva Geneva Switzerland; ^9^ Jiangsu Key Laboratory of Molecular Medicine Nanjing Jiangsu China; ^10^ Institute of Brain Science Nanjing University Nanjing Jiangsu China

**Keywords:** fetal MRI, high dielectric constant, image quality, specific absorption rate

## Abstract

**Background:**

At high magnetic fields, degraded image quality due to dielectric artifacts and elevated specific absorption rate (SAR) are two technical challenges in fetal MRI.

**Purpose:**

To assess the potential of high dielectric constant (HDC) pad in increasing image quality and decreasing SAR for 3 T fetal MRI.

**Study Type:**

Prospective.

**Field Strength/Sequence:**

3 T. Balanced steady‐state free precession (bSSFP) and single‐shot fast spin‐echo (SSFSE).

**Population:**

One hundred twenty‐eight participants (maternal‐age 29.0 ± 3.6, range 20–40; gestational‐age 30.3 ± 3.5 weeks, range 22–37 weeks) undertook bSSFP and 40 participants (maternal‐age 29.5 ± 3.8, range 19–40; gestational‐age 30.4 ± 3.5 weeks, range 23–37 weeks) undertook SSFSE.

**Assessment:**

Patient clinical characteristics were recorded, such as gestational‐age, amniotic‐fluid‐index, abdominal‐circumference, body‐mass‐index, and fetal‐presentation. Quantitative Image‐quality analysis included signal‐to‐noise ratio (SNR) and contrast‐to‐noise ratio (CNR). Qualitative analysis was performed by three radiologists with four‐point scale to evaluate overall image quality, dielectric artifact, and diagnostic confidence. Whole‐body total SAR was obtained from the vendor workstation.

**Statistical Testing:**

Paired rank sum test was used to analyze the differences in SNR, CNR, overall image quality, dielectric artifact, diagnostic confidence, and SAR with and without HDC pad. Spearman correlation test was used to detect correlations between image quality variable changes and patient clinical characteristics. *P* values <0.05 were set as statistical significance.

**Results:**

With HDC pad, SNR and CNR was significantly higher (41.45% increase in SNR, 54.05% increase in CNR on bSSFP; 258.76% increase in SNR, 459.55% increase in CNR on SSFSE). Overall qualitative image quality, dielectric artifact and diagnostic confidence improved significantly. Adding HDC pad significantly reduced Whole‐body total SAR (32.60% on bSSFP; 15.40% on SSFSE). There was no significant correlation between image quality variable changes and participant clinical characteristics (*P*‐values ranging from 0.072 to 0.992).

**Data Conclusion:**

In the clinical setting, adding a HDC pad might increase image quality while reducing dielectric artifact and SAR.

**Plan Language Summary:**

Dielectric artifacts and elevated SAR are two technical problems in 3T fetal MRI. In a prospective analysis of 168 pregnant participants undertaking 3.0T fetal MRI scanning, high dielectric constant (HDC) pad increased SNR by 41.45%, CNR by 54.05% on bSSFP, and SNR by 258.76%, CNR by 459.55% on SSFSE. Overall image quality, dielectric artifact reduction, and diagnostic confidence assessed by three radiologists was improved. Whole‐body total SAR decreased by 32.60% on bSSFP and by 15.40% on SSFSE. These findings suggested that the HDC pad can enhance fetal MRI safety and quality, making it a promising tool for clinical practice.

**Evidence Level:**

2

**Technical Efficacy:**

Stage 5

Fetal MRI is being increasing used for prenatal diagnosis of abnormalities.^1^, ^2^ Although ultrasound (US) remains the modality of choice for evaluating fetal anatomy and screening the general population, MRI offers much greater detail with higher soft tissue resolution and contrast than US.^3^

In recent years, many hospitals have shifted to performing fetal MRI at 3 T.^4^, ^5^ The high signal‐to‐noise ratio (SNR) achieved at 3 T enables increased spatial resolution and decreased acquisition time.^6^ However, several technical challenges arise at higher static magnetic field. Radio‐frequency magnetic (*B*_1_^+^) field inhomogeneities are more pronounced at 3 T, leading to dielectric artifacts.^7^ This occurs because the wavelength of the transmission field is shortened at 3 T and becomes comparable to the dimensions of the gravid abdomen.^8^ The resulting artifact causes local areas of signal loss, manifested as shading, which degrades image quality.

Another concern in fetal MRI is the potential impact of radiofrequency power deposition on both the fetus and the mother, especially given the high conductivity of the amniotic fluid.^9^, ^10^ According to the International Electrotechnical Commission standards, the allowed specific absorption rate (SAR) is limited to a whole‐body averaged SAR of 2 W/kg. Under the same imaging conditions, SAR at 3 T is higher than 1.5 T.^11^ If the regulatory limits are reached during the scanning, image parameters such as flip angles and repetition time need to be adjusted, which prolongs acquisition time and compromises image quality.^12^

In previous electromagnetic simulation studies, high dielectric constant (HDC) pads have shown the potential to enhance *B*_1_^+^ field homogeneity and reduce SAR in 3 T MRI scanning.^12^, ^13^, ^14^ The primary effect of dielectric materials is to decrease the wavelength of radiofrequency field. Luo et al reported a 24.75% decrease in SAR after adding HDC pads.^15^ Ruppert et al revealed that there was over 50% increase in SNR and strong transmission power reduction with HDC pads.^16^ HDC pads have the potential to increase imaging sensitivity and promote safety.^17^ In previous simulation study, the HDC pad could improve *B*_1_^+^ field efficiency and reduce SAR in fetal head area.^18^ These features are particularly beneficial for fetal MRI. However, these researches were performed on phantoms with electromagnetic filed simulation. The effects of HDC pads on MRI scanning of pregnant women at 3 T remain unclear.

Currently, single‐shot fast spin‐echo (SSFSE) and balanced steady state free precession (bSSFP) are two commonly used *T*_2_‐weighted fetal MRI sequences.^19^ This study aimed to assess whether adding a HDC pad in 3 T fetal MRI can increase image quality and decrease SAR.

## Materials and Methods

### Study Population

This prospective single‐center observational study was approved by the local institutional review board (2022‐141‐01). Informed consent was acquired from all participants prior to MRI scanning.

**FIGURE 1:**
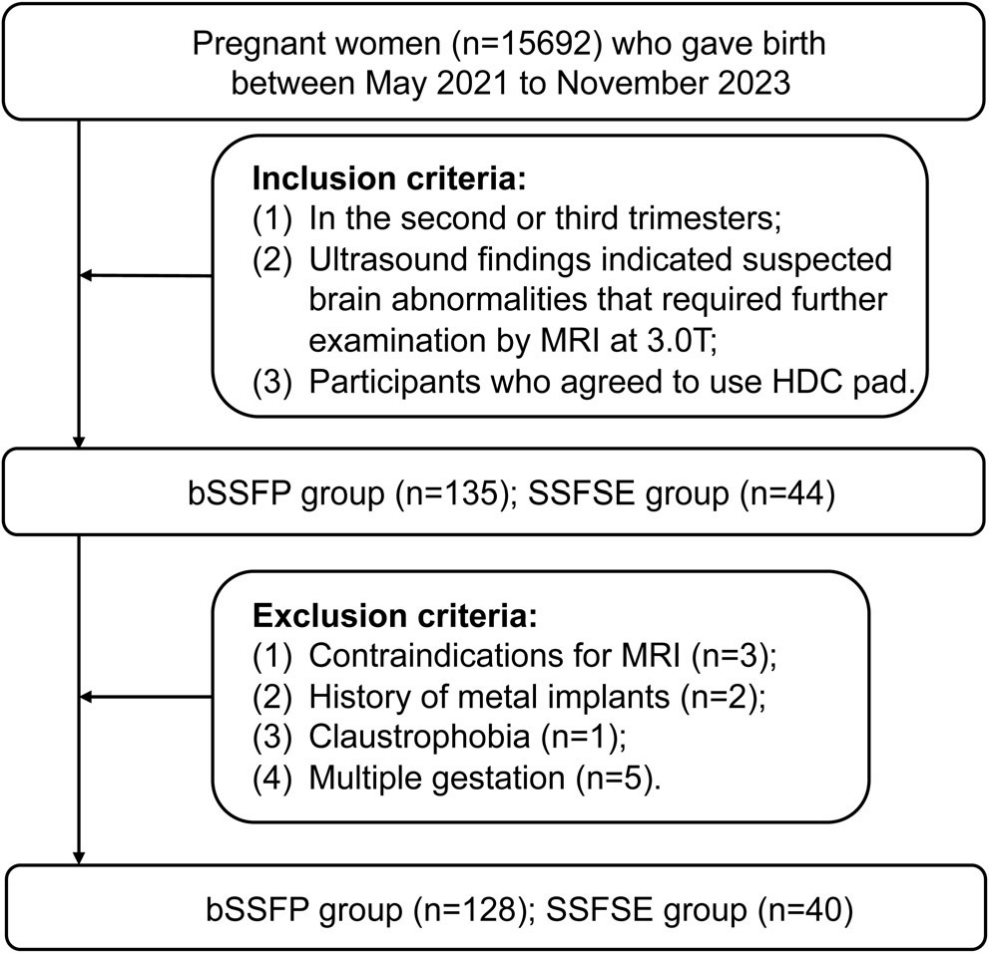
Participant selection flowchart. HDC = high dielectric constant; bSSFP = balanced steady state free precession; SSFSE = single‐shot fast spin‐echo.

This research involved pregnant participants who gave birth between May 1, 2021 and November 31, 2023. Inclusion criteria: 1) Pregnant women in the second or third trimesters; 2) US findings indicated suspected brain abnormalities that required further examination by MRI; 3) Participants who agreed to use HDC pad. Exclusion criteria: 1) Contraindications for MRI; 2) History of metal implants; 3) Claustrophobia; 4) Multiple gestation. Participant selection flowchart are illustrated in Fig. 1. Participants' clinical characteristics were recorded, including maternal age, gestational age (GA), abdominal circumference (AC), amniotic fluid index (AFI), body mass index (BMI), and fetal presentation.

### HDC Pad Design

**FIGURE 2:**
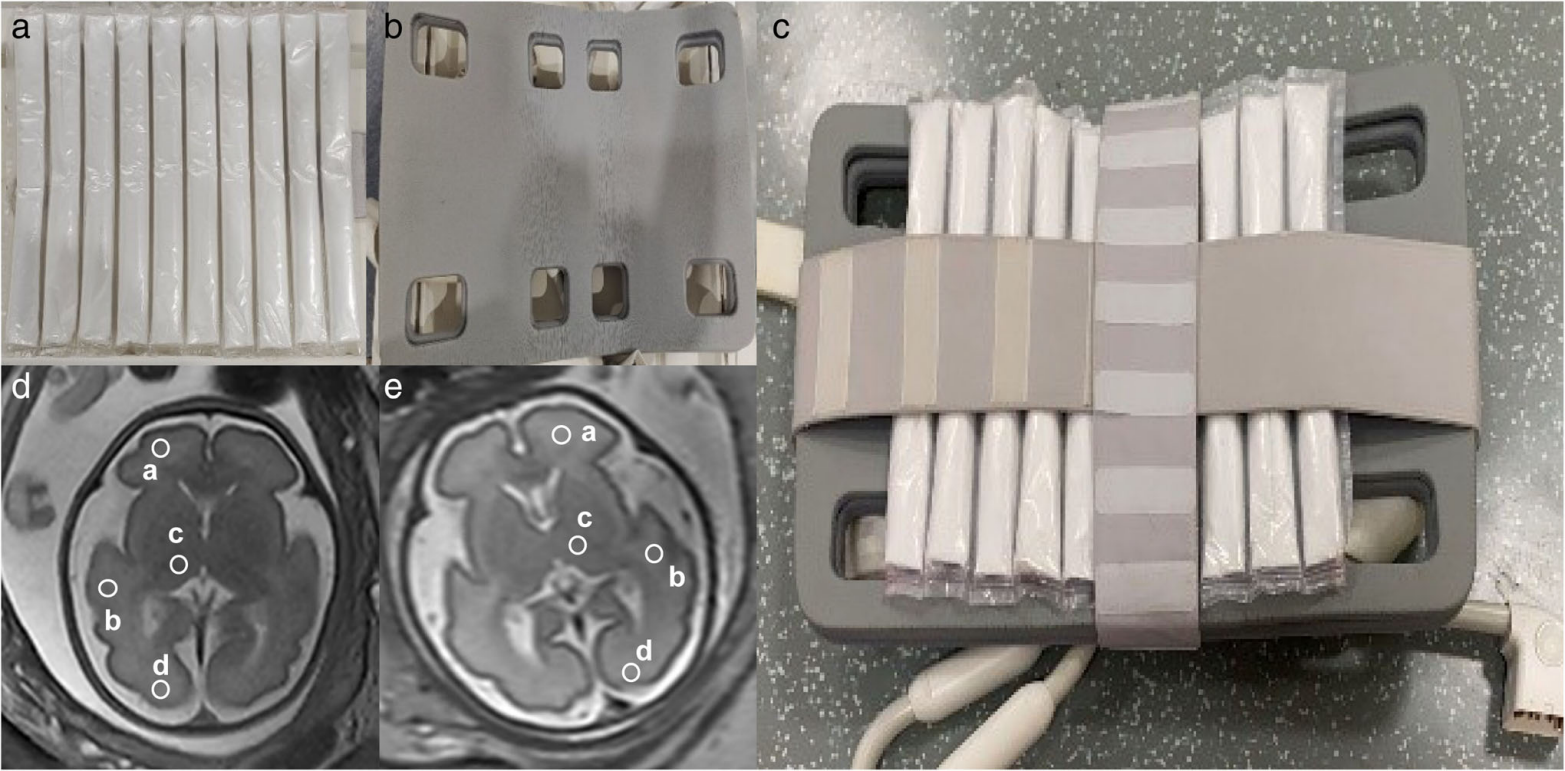
(**a**) The home‐made HDC pad as used experimentally with 10 compartments, each filled with BaTiO_3_ granules mixed with D_2_O with the ratio of 4:1. (**b**) Clinically used abdominal coil. (**c**) HDC pad tied to the inside of the abdominal coil. (**d**) ROI example for bSSFP sequence. (**e**) ROI example for SSFSE sequence. frontal lobe (a), temporal lobe (b), thalamus (c), occipital lobe (d). ROIs were placed on the side close to the abdominal wall. bSSFP = balanced steady state free precession; SSFSE = single‐shot fast spin‐echo.

The HDC material was designed as a dense mixture of barium titanate (BaTiO_3_) to Deuterium oxide (D_2_O) with a ratio of 4:1. We filled HDC material into 10 plastic bags measuring 350 mm × 40 mm × 15 mm, ensuring as little air as possible within the bags. The HDC bags were then arranged into vertical strip‐like partitions to achieve a uniform distribution of HDC material. We sealed 10 HDC bags together to create a HDC pad (Fig. 2). The relative permittivity and the electric conductivity of the pad was measured by a dielectric probe kit (DAK12‐TL, Schmid & Partner Engineering AG, Zurich, Switzerland). This single pad was used for the whole study.

### MRI Acquisition Protocol

Data were obtained using 3 T MRI scanners. SSFSE images were acquired on uMR770 and bSSFP Images were acquired on uMR790 (United Imaging Healthcare, Shanghai, China). The scanning parameters for SSFSE sequence is as follows: repetition time (TR)/echo time (TE), 2000/4.68 msec; slice thickness, 3 mm; flip angle, 140°; field of view (FOV), 380 mm × 350 mm; number of slices, 35; number of signal averaged (NSA), 2; matrix, 288 × 80; acquisition time, 37 s. The scanning parameters for bSSFP sequence is as follows: TR/TE, 4.68/2.35 msec; slice thickness, 3 mm; flip angle, 100°; FOV, 330 mm × 330 mm; number of slices, 36; NSA, 1; matrix, 256 × 80; acquisition time, 70 s. Parallel imaging was not used. All participants were first scanned in axial, coronal and sagittal plane with HDC pad for clinal diagnosis. Then participants were repositioned to the same location and rescanned with the pad removed in axial plane. Forty‐six participants were also scanned in the coronal and sagittal plane without pad. Two‐channel radiofrequency shimming was set during the scanning. The transmission voltages were automatically adjusted by the machines. Thirty‐seven acquisitions were repeated because severe fetal motion was encountered. After evaluation of motion artifacts by C.Y. with 10 years of experience, only axial images without severe motion artifacts were selected for further analysis.

During the scanning, the participants' condition was monitored with frequent verbal interaction to identify potential vasovagal episode. Twenty‐five participants were asked to evaluate comfort with and without HDC pad on a scale: 1) (significant discomfort, participants cannot tolerant the scan); 2) (moderate discomfort, participants require pauses but can still complete the scan); 3) (mild discomfort, participants can complete the scan without pauses); 4) (no discomfort, participants can complete the scan comfortably).

### Quantitative Image Analysis

Three radiologists C.Y. with 10 years of experience, Z.W. with 12 years of experience and X.Z. with 15 years of experience independently performed image analysis. Four Regions of interest (ROI) measuring between 200 and 400 mm^2^ were placed on the axial section of each fetal brain. The ROIs contained four anatomical structures: frontal lobe, temporal lobe, thalamus, and occipital lobe (Fig. 2). The four ROIs were placed on the side close to the abdominal wall. SNR and contrast‐noise‐ratio (CNR) were calculated according to the following formulas:

$$\mathrm{SNR}=\frac{S_{\mathrm{ROI}}}{\text{noise}}.$$

$$\mathrm{CNR}=\frac{\left|S_{\mathrm{ROI}}-S_{\text{muscle}}\right|}{\text{noise}}.$$

*S*_ROI_ was defined as the mean value of the signal from the ROI. *S*_muscle_ was defined as the mean value of the signal from abdominal wall muscle. Noise was estimated by measuring the signal standard deviation within the imaging background.

### Image Analysis

Image analysis was also conducted by the abovementioned three radiologists. The image assessment consisted of each reader reviewing one group of 15–20 examinations at a day. These groups were mixed of images obtained with and without HDC pad in a random order. To limit recall bias, interpretations for each participant's images with and without HDC pad were separated with a minimum 4‐week washout period. The readers were blinded to the pad status, radiologic reports, and each other's evaluations. All the sequence and participant‐identifying markers were removed. The readers could only evaluate images without annotations but had access to the clinical indications because they could obtain this information in daily clinical routine. All readers evaluated images utilizing a four‐point Likert scale to define overall image quality, dielectric artifact, and diagnostic confidence. Overall image quality: 1) (poor image quality, indistinct delineation with disturbed internal structure); 2) (fair image quality, suboptimal anatomic delineation); 3) (good image quality, well delineated anatomic structures); 4) (excellent image quality, excellent delineated and contrasted structures). Dielectric artifacts: 1) (severe impediment of image quality by dielectric artifact); 2) (moderate impediment of image quality by dielectric artifact); 3) (mild impediment of image quality by dielectric artifact); 4) (no visible dielectric artifact). Diagnostic Confidence: 1) (nondiagnostic, rescanning necessary); 2) (image quality with diagnostic limitations); 3) (image quality without relevant diagnostic limitations); 4) (excellent image quality without any diagnostic limitations).

### Specific Absorption Rate

The whole‐body total SAR of the images was recorded on United Imaging software workstation.

### Statistical Analysis

The Kolmogorov–Smirnov test was used to assess whether the data were following normal distribution. The Wilcoxon signed rank test was used to test the difference of SNR, CNR, overall image quality, dielectric artifact diagnostic confidence SAR of images with and without HDC pad. For each sequence, we utilized Bonferroni correction for multiple comparisons of all the values of SNR, CNR, and SAR. Spearman correlation test was used to detect correlations between image quality variable changes and patient clinical characteristics. Fleiss *κ* values were used to assess the intraobserver and interobserver agreement. The strength of agreement for *κ* values is as follows: 0–0.20, poor; 0.21–0.40, fair; 0.41–0.60, moderate; 0.61–0.80, substantial; 0.81–1.00, almost perfect.

The threshold for statistical significance was set at *P* < 0.05. Statistical analysis was executed using SPSS (version 26.0, IBM, USA).

## Results

### Participant Characteristics

A total of 168 participants were recruited in this research, of which 128 participants were scanned with bSSFP and 40 participants were scanned with SSFSE. The baseline characteristics and detailed participants' conditions of these two groups are shown in Table 1.

**TABLE 1:** Patient Characteristics According to *T*_2_‐Weighted Sequences

Variables	bSSFP (N = 128)	SSFSE (N = 40)	*P*‐Value
Maternal age (years)	29.51 ± 3.81 (range, 19–40)	29.08 ± 3.55 (range, 20–40)	0.409
GA (weeks)	30.25 ± 3.53 (range, 22–37)	30.38 ± 3.50 (range, 23–37)	0.839
Second trimester	30	11	
Third trimester	98	29	
Fetal presentation			0.871
Vertex	107	33	
Breech	21	7	
AC	95.28 ± 7.88	‐	‐
AFI	137 (120, 157)	‐	‐
BMI	25.37 (23.31, 28.24)	‐	‐
No. of normal fetal brain	50	22	
No. of abnormalities	78	18	
Ventriculomegaly	33	8	
Midline cyst	16	2	
Posterior cerebral fossa cistern enlargement	12	6	
Septum pellucidum abnormality	6	0	
Corpus callosum abnormality	2	0	
Head smaller than fourth centile	3	0	
Tuberous sclerosis	0	1	
Lissencephaly	1	0	
Intracranial bleeding	1	0	
Cerebellar vermis dysplasia	0	1	
Malformation of cortical development	1	0	
Polymicrogyria	1	0	
Other abnormalities of uncertainty	2	0	

bSSFP = balanced steady state free precession; SSFSE = single‐shot fast spin‐echo; GA = gestational age; AC = abdominal circumference; AFI = amniotic fluid index; BMI = body mass index.

The survey regarding participant comfort resulted in a median value of 3 (3, 4) for scanning without HDC pad compared with 3 (3, 4) for scanning with HDC pad (*P* = 0.18).

### Dielectric Properties and Conductivity of the HDC Pad

The relative permittivity and the electric conductivity of the HDC pad were 223.699 and 0.372 S/m, respectively.

### Interobserver and Intraobserver Agreements

Interobserver agreements for SNR ranged from 0.728 to 0.911. Interobserver agreements for CNR ranged from 0.701 to 0.943. Intraobserver agreements for SNR ranged from 0.804 to 0.945. Intraobserver agreements for SNR ranged from 0.726 to 0.873 (Table S1 in the Supplemental Material).

### Comparison of Quantitative Evaluation

**FIGURE 3:**
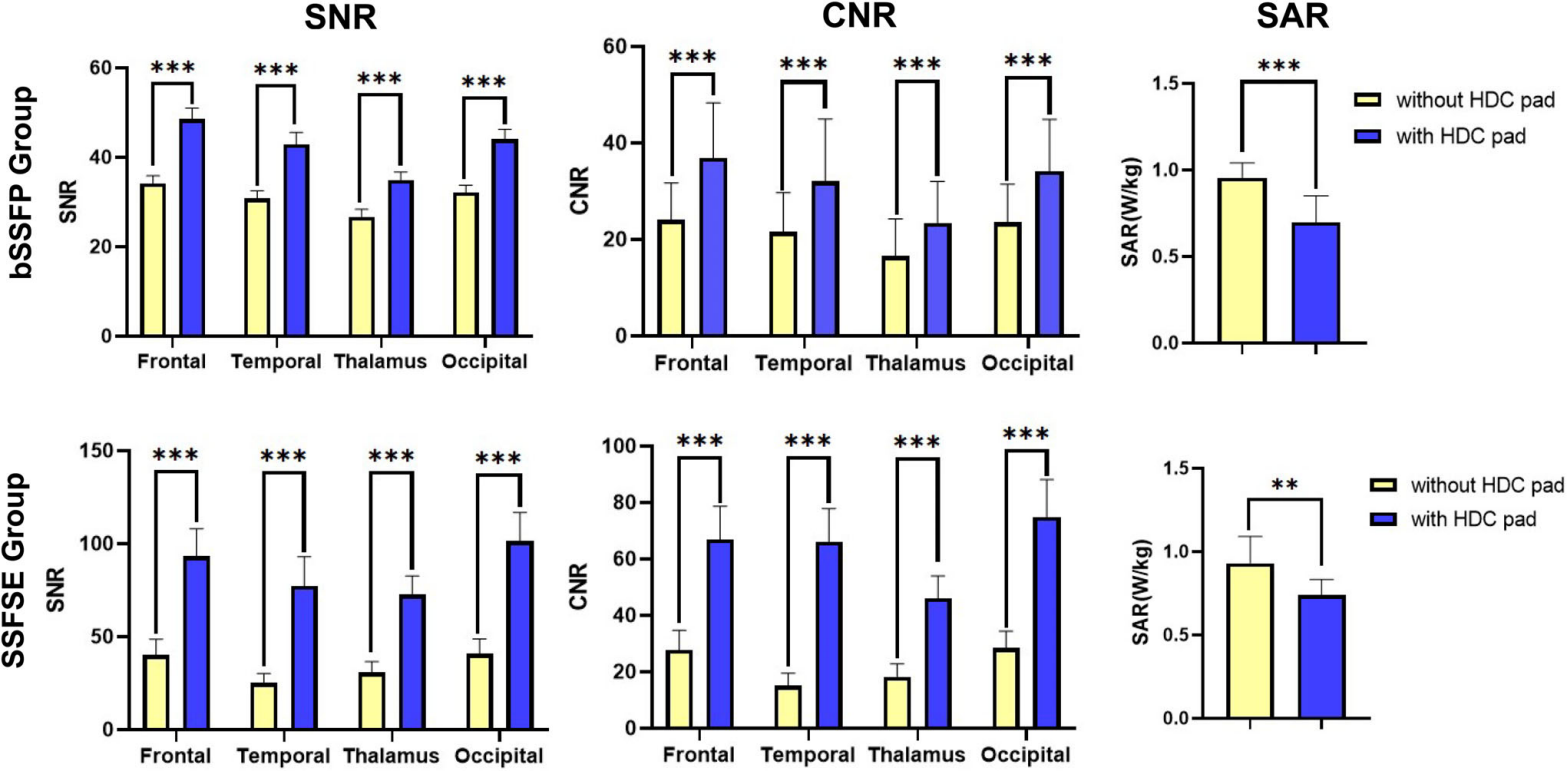
Comparation of SNR, CNR, and SAR of bSSFP (N = 128) and SSFSE (N = 40) sequences with and without HDC pad. bSSFP = balanced steady state free precession; SSFSE = single‐shot fast spin‐echo; SNR = signal‐to‐noise ratio; CNR = contrast‐to‐noise ratio; SAR = specific absorption rate; HDC = high dielectric constant. **P < 0.01; ***P < 0.001.

As indicated in Table 2 and Fig. 3, SNR and CNR of frontal lobe, temporal lobe, thalamus, and occipital lobe all increased significantly with the HDC pad on bSSFP and SSFSE sequences.

**TABLE 2:** Image Quality Variables and SAR of bSSFP and SSFSE Images Without and With HDC Pad

Variables	ROI	bSSFP		*P*‐Value	SSFSE		*P*‐Value
		Without HDC Pad	With HDC Pad		Without HDC Pad	With HDC Pad	
SNR	Frontal	32.44 (27.03, 38.85)	47.10 (36.68, 55.35)	<0.001	36.28 (21.75, 52.27)	93.47 (55.40, 117.92)	<0.001
	Temporal	29.91 (24.52, 35.53)	40.34 (32.24, 49.57)	<0.001	19.79 (13.95, 33.12)	78.00 (46.03, 95.66)	<0.001
	Thalamus	25.07 (20.70, 30.16)	33.03 (27.58, 40.33)	<0.001	30.16 (20.40, 37.97)	78.00 (46.03, 95.66)	<0.001
	Occipital	31.42 (26.06, 38.79)	42.50 (35.24, 52.89)	<0.001	39.40 (27.27, 48.83)	86.31 (65.32, 138.88)	<0.001
CNR	Frontal	23.11 (18.48, 27.60)	35.57 (28.75, 42.10)	<0.001	23.81 (12.08, 38.10)	67.34 (35.48, 87.40)	<0.001
	Temporal	21.02 (16.45, 25.25)	28.69 (23.39, 38.53)	<0.001	10.78 (5.84, 22.78)	59.69 (39.20, 80.06)	<0.001
	Thalamus	15.30 (12.00, 19.75)	22.08 (16.80, 27.98)	<0.001	17.20 (7.38, 24.71)	46.71 (26.76, 59.78)	<0.001
	Occipital	23.03 (18.17, 28.69)	32.75 (26.90, 41.17)	<0.001	28.26 (18.10, 34.98)	61.75 (38.02, 112.69)	<0.001
Overall image quality	‐	2 (2, 3)	3 (3, 4)	<0.001	3 (2, 3)	4 (3, 4)	<0.001
Dielectric artifact	‐	2 (2, 3)	3 (3, 4)	<0.001	3 (2, 3)	4 (3, 4)	<0.001
Diagnostic confidence	‐	3 (2, 3)	3 (3, 4)	<0.001	3 (3, 4)	4 (3, 4)	<0.001
SAR	‐	0.95 (0.89, 1.16)	0.68 (0.60, 0.76)	<0.001	0.80 (0.53, 1.08)	0.68 (0.51, 0.87)	0.005

bSSFP = balanced steady state free precession; SSFSE = single‐shot fast spin‐echo; ROI = region of interests; HDC = high dielectric constant; SNR = signal‐to‐noise ratio; CNR = contrast‐to‐noise ratio; SAR = specific absorption rate.

For bSSFP sequence, after adding HDC pad, SNR increased by 46%, 43%, 35%, and 42% respectively on frontal lobe, temporal lobe, thalamus, and occipital lobe. CNR increased by 59%, 55%, 48%, and 54% respectively on four ROIs. On average, SNR increased by 41% and CNR increased by 54% with HDC pad.

For SSFSE sequence, after adding HDC pad, SNR increased by 246%, 305%, 233%, and 252% respectively on four ROIs. CNR increased by 433%, 715%, 496%, and 315% respectively on four ROIs. On average, SNR increased by 259% and CNR increased by 460% with HDC pad.

### Comparison of Qualitive Evaluation

**FIGURE 4:**
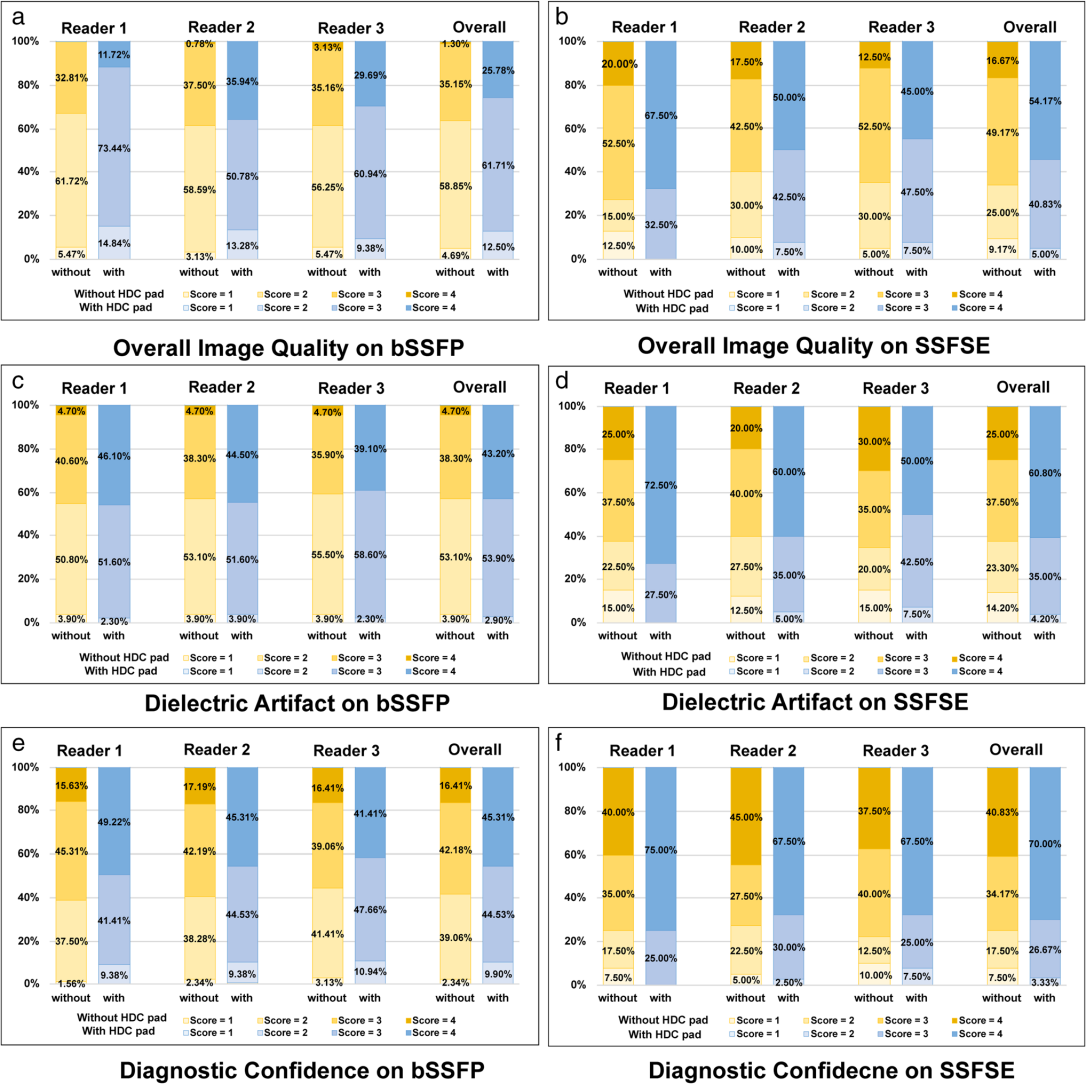
Bar graphs show the results of the analysis of overall image quality (**a**, **b**), dielectric artifact (**c**, **d**) and diagnostic confidence (**e**, **f**) for bSSFP and SSFSE images with and without HDC pad. bSSFP = balanced steady state free precession; SSFSE = single‐shot fast spin‐echo.

**FIGURE 5:**
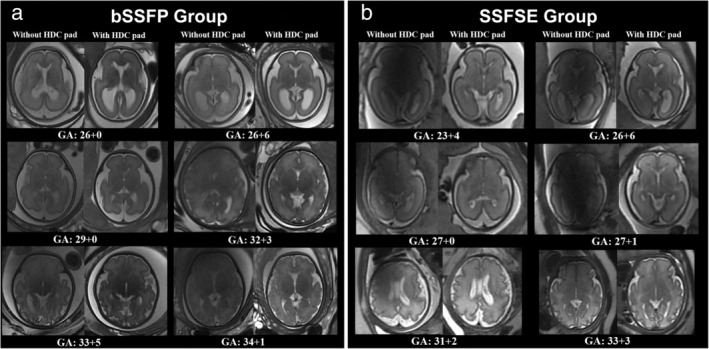
(**a**) Transverse bSSFP images of fetal brain of different GA with and without HDC pad. (**b**) Transverse SSFSE images of fetal brain of different GA without and with HDC pad. bSSFP = balanced steady state free precession; SSFSE = single‐shot fast spin‐echo; GA = gestational age.

**FIGURE 6:**
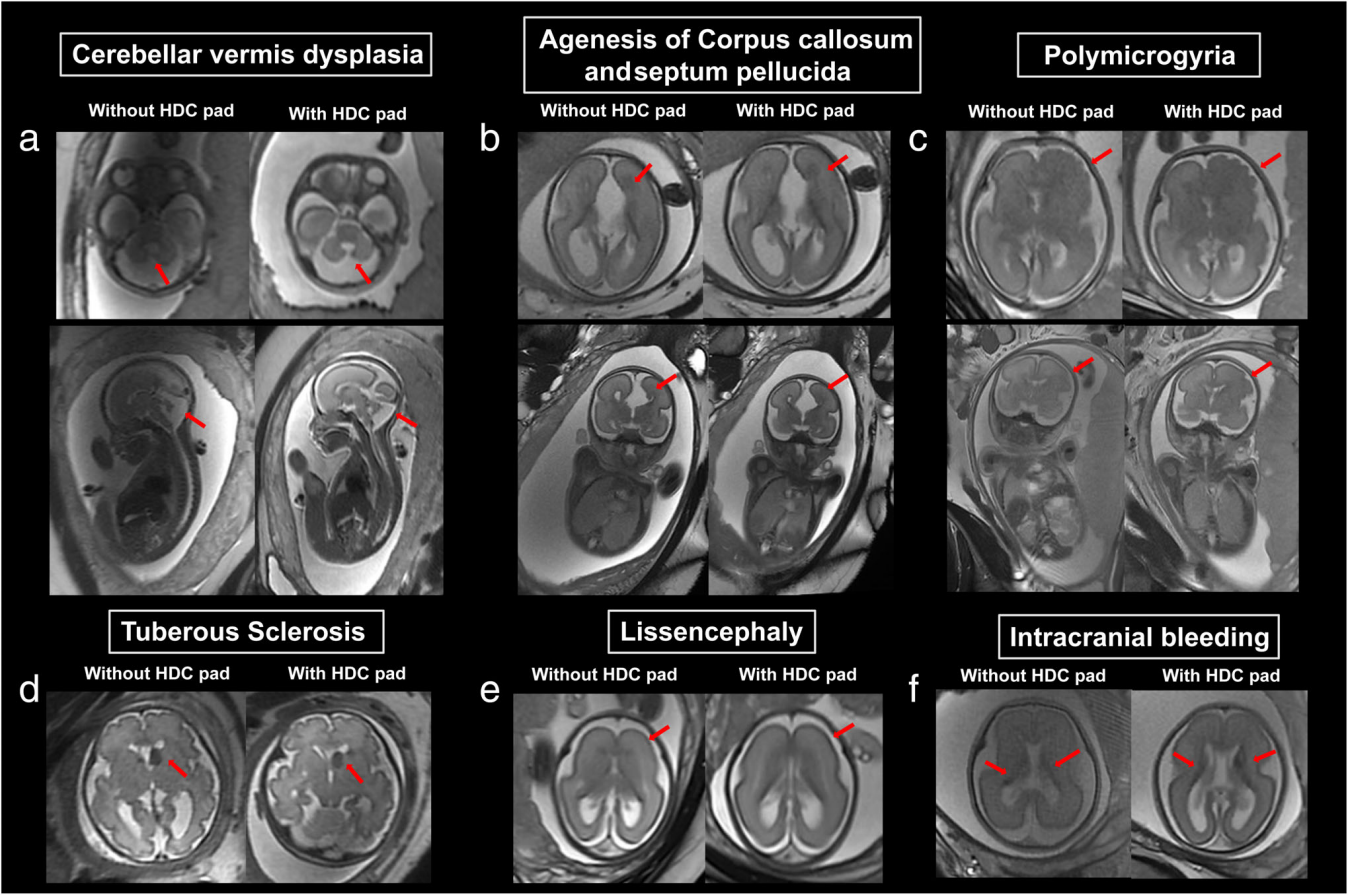
Images of fetal brain of different abnormalities without and with HDC pad. (**a**) Axial and sagittal SSFSE images of cerebellar vermis dysplasia (GA, 23 + 6 weeks). (**b**) Axial and coronal bSSFP images of agenesis of corpus callosum and septum pellucida (GA, 25 + 3 weeks). (**c**) Axial and coronal bSSFP images of polymicrogyria (GA, 26 + 0 weeks). (**d**) Axial SSFSE images of tuberous sclerosis (GA, 30 + 3 weeks). (**e**) Axial bSSFP images of lissencephaly (GA, 35 + 2 weeks). (**f**) Axial SSFSE images of intracranial bleeding (GA, 24 + 0 weeks). bSSFP = balanced steady state free precession; SSFSE = single‐shot fast spin‐echo; GA = gestational age.

After adding HDC pad, for the bSSFE sequence, Overall image quality improved significantly from 2 (2, 3) to 3 (3, 4); Dielectric artifact improved significantly from 2 (2, 3) to 3 (3, 4); Diagnostic confidence improved significantly from 3 (2, 3) to 3 (3, 4). For the SSFSE sequence, Overall image quality improved significantly from 3 (2, 3) to 4 (3, 4); Dielectric artifact improved significantly from 3 (2, 3) to 4 (3, 4); Diagnostic confidence improved significantly from 3 (3, 4) to 4 (3, 4), as shown in Fig. 4. Figure 5 shows transverse bSSFP and SSFSE images of fetal brain of different GA with and without the HDC pad, respectively. Figure 6 shows images of fetal brain of different neurologic abnormalities.

### Comparison of SAR Value

After adding HDC pad, whole‐body total SAR significantly decreased by 33% on bSSFP sequence and by 15% on SSFSE sequence.

### Correlation between Image Quality Changes and Participant Clinical Characteristics

For the bSSFP sequence, the changes of SNR, CNR, overall image quality, dielectric artifact and diagnostic confidence illustrated no significant correlations with participants' clinical characteristics including GA (*P*‐values ranging from 0.082 to 0.905), AFI (*P*‐values ranging from 0.085 to 0.615), AC (*P*‐values ranging from 0.260 to 0.780), BMI (*P*‐values ranging from 0.072 to 0.959), and fetal presentation (*P*‐values ranging from 0.281 to 0.983). For SSFSE sequence, the changes of the abovementioned metrics illustrated no significant correlations with participants' GA (*P*‐values ranging from 0.231 to 0.992) and fetal presentation (*P*‐values ranging from 0.162 to 0.846).

## Discussion

In this research, we evaluated effects of HDC pad on 3 T fetal MRI scanning in clinical scenario beyond electromagnetic simulations on phantoms. This study illustrated enhanced SNR and tissue contrast, as well as reduced SAR on both bSSFP and SSFSE sequences. The results demonstrated HDC pad possessed the potential for improving imaging quality and patient safety of 3 T fetal MRI and performed well in pregnant women with different GA, AC, AFI, BMI, and fetal presentation.

Before the clinical application of HDC pad, we conducted a stimulation study and discovered that HDC pad could improve local *B*_1_^+^ field efficiency by 16.6%, reduce local SAR of fetal head by 34.4% and reduce whole‐body total SAR by 7.7%.^18^ The effects were more obvious when phantom was placed on left lateral position.

Fetal MRI is an important diagnostic imaging technique adjunct to US, especially for detection and evaluation for abnormalities of fetal brain, lungs, spine and bowel and the placenta.^20^, ^21^, ^22^ When fetal MRI first became widely used in clinical practice, it was primarily performed on 1.5 T because the safety of fetal MRI at 3 T was not well established.^23^ Several literature examining the safety profile of 3 T fetal MRI has been promising.^24^ Some researchers examining long‐term postnatal outcomes of fetuses who underwent 3.0 T MRI reported no adverse impact on fetal growth and neonatal hearing.^25^, ^26^ Three T fetal MRI has become more available in recent years.^27^, ^28^ Therefore, increasing image quality and safety of 3 T fetal MRI is of great importance.

SNR and CNR in MRI are roughly linearly proportional to magnetic field strength. However, due to *B*_1_^+^ field inhomogeneities, image quality may be degraded at higher magnetic field.^29^ The problem is especially more severe during pregnancy because of the high conductivity of amniotic fluid and increased abdominal size. Yetisir et al revealed that radiofrequency shimming can improve imaging compared to birdcage mode without increasing fetal and maternal SAR when a patient‐specific SAR model is incorporated into the shimming procedure.^9^ Many methods can be used for shimming, such as parallel transmission and improved coil design.^30^, ^31^ Adding a HDC pad is another method for shimming. Kataoka et al applied HDC pad for 3 T pelvic MR scanning of healthy females, which improved image homogeneity and reduced dielectric artifact.^32^ Our results were consistent with previous simulation research, revealing that SNR, CNR, and image quality of pregnant participants increase significantly after adding a HDC pad.

SAR and heating are crucial factors for 3 T fetal MRI. Increased temperature experienced by the gravid participants for relatively prolonged periods may be harmful and teratogenic.^33^ Clinically, it is ideal to keep the energy delivered to pregnant patients as low as possible. The SAR values analyzed in our study were average whole‐body total SAR automatically calculated by the MRI vendors software. Due to technical limitations, we could not conduct further analysis to compare the radiofrequency power deposited on the uterus, whole fetal body, and fetal brain separately. Local SAR can potentially exceed limits even when global SAR remains within safety thresholds, particularly in anatomically complex regions like gestational uterus. In a previous stimulation study, whole‐body total SAR was reduced by up to 31% with HDC pad in pregnant subjects at the 3rd, 7th, and 9th month of gestation.^17^ Another research reported increases in heating radiofrequency of up to 49% was detected in body phantom after removing HDC pad.^12^ HDC pad can enhance *B*_1_^+^ field efficiency and homogeneity, resulting in a diminished input power requirement and reduced local SAR and whole‐body total SAR.^34^, ^35^, ^36^ Previous research reported that some clinically used 3 T sequences with relatively high SAR, such as 2D *T*_1_‐weighted spoiled gradient‐echo and 3D bSSFP, may need modification to restrict the energy deposited on the patient.^37^ Adding a HDC pad can provide a practical way to make these high‐SAR sequences safer and more applicable. The decrease of SAR value is beneficial for controlling local temperature elevation and safety for both mother and fetus.

The SNR enhancement and SAR reduction accomplished in this research were realized with a home‐made HDC pad composed of dense mixture of 4:1 BaTiO_3_ to D_2_O, which is similar to previous research.^14^, ^17^ The primary effect of dielectric materials is to decrease the wavelength of radiofrequency field. Most normal tissues in human body have a dielectric constant of 50, which shortens the wavelength of manufactures at 3 T from 235 centimeters in free space to 33 centimeters in normal tissues. This is similar to the FOV for fetal imaging, thus isolated areas of disruptive interference can commonly occur inside 3 T fetal imaging. The HDC material has a higher dielectric constant and subsequently shortens wavelength than normal body tissues, eliminating the destructive dielectric artifacts in fetal MRI.

The geometry of pad placement may influence the effects of image quality improvement. In this research, the flat HDC pad was positioned with its orientation parallel to the maternal body's long axis. This alignment was chosen to optimize the interaction between the dielectric material and the radiofrequency field. The parallel orientation also aligns naturally with the anatomical contours of the maternal abdominal wall, increasing placement stability and minimizing variability between participants. Even though the current geometric design and placement strategy of the pad in our study exhibited effective image quality improvement and SAR reduction, we acknowledge that the refinement of pad geometry, size and orientation could potentially lead to better results by more effectively adapting to different maternal anatomies and imaging conditions. We expect greater improvements can be achieved after further investigation of optimizing dielectric material and geometric design of the pad.

### Limitations

The HDC pad used in this study was home‐made, which limits the reproducibility of the results. Second, imaging data was collected from a single center. Third, we only conducted experiments on magnetic resonance machines produced by United Imaging and did not include machines from other vendors. Lastly, we only assessed two *T*_2_‐weighted structural sequences SSFSE and bSSFP. Whether HDC pad can improve image quality for other sequences needs further exploration in the future.

## Conclusion

Adding a HDC pad can increase overall quantitative and qualitative image quality while reducing dielectric artifact and SAR in a clinical setting.

## Supporting information


**Data S1:** Supporting Information.
