# Supplementary material for: Improving Image Quality and Decreasing SAR With High Dielectric Constant Pads in 3 T Fetal MRI
Source: J Magn Reson Imaging. 2025 Jan 21;61(6):2505–15. doi: 10.1002/jmri.29677 (PMC12063766; doi:10.1002/jmri.29677)
Supplement: Supplementary file 1 — Data S1: Supporting Information. [file JMRI-61-2505-s001.docx]

**Supplementary Materials**

Power calculation

Due to lack of previous clinical research using HDC pad on fetal MRI scanning, we didn’t conduct power analysis and sample size estimation before commencing this research. Alternatively, we performed post-hoc power analysis. The SNR, CNR and SAR values in our research didn’t meet the normality assumptions necessary for parametric tests, we utilized Wilcoxon signed-rank test. To estimate the effect size, we calculated the standardized test statistic from the Wilcoxon signed-rank test. The effect size (r) was computed using the formula r=Z/√N, where Z is the standardized test statistic and N is the number of paired observations. A significance level (α) was set at 0.05 and a desired power (1 – β) was set at 0.80. For bSSFP sequence, sample sizes of 12, 12, 11 were required for SNR, CNR and SAR, respectively. For SSFSE sequence, sample sizes of 13, 14, 35 were required for SNR, CNR and SAR, respectively. The sample size of 128 in bSSFP group and 40 in SSFSE group in this research ensured the robustness of our statistical conclusions.

Table S1 Inter-observer reproducibility for measurements of SNR and CNR on different ROIs

|  | Variables | ROI | Interobserver agreement (95% CI) | Intraobserver agreement (95% CI) |
| --- | --- | --- | --- | --- |
| without HDC pad | SNR | Frontal | 0.784(0.694~0.848) | 0.814 (0.736~0.829) |
|  |  | Temporal | 0.863(0.805~0.903) | 0.930 (0.877~0.954) |
|  |  | Thalamus | 0.911(0.874~0.937) | 0.822 (0.753~0.846) |
|  |  | Occipital | 0.792(0.705~0.854) | 0.867 (0.820~0.919) |
|  | CNR | Frontal | 0.843(0.778~0.890) | 0.726 (0.689~0.871) |
|  |  | Temporal | 0.887(0.840~0.921) | 0.869 (0.814~0.916) |
|  |  | Thalamus | 0.943(0.920~0.960) | 0.774 (0.684~0.835) |
|  |  | Occipital | 0.839(0.772~0.887) | 0.823 (0.767~0.871) |
| with HDC pad | SNR | Frontal | 0.842 (0.776~0.889) | 0.900 (0.889~0.949) |
|  |  | Temporal | 0.902(0.861~0.961) | 0.945 (0.898~0.978) |
|  |  | Thalamus | 0.832(0.762~0.882) | 0.804 (0.753~0.926) |
|  |  | Occipital | 0.728(0.598~0.820) | 0.856 (0.784~0.922) |
|  | CNR | Frontal | 0.701(0.668~0.866) | 0.873 (0.815~0.932) |
|  |  | Temporal | 0.920(0.886~0.943) | 0.852 (0.796~0.920) |
|  |  | Thalamus | 0.878(0.827~0.914) | 0.770 (0.703~0.835) |
|  |  | Occipital | 0.781(0.617~0.810) | 0.814 (0.758~0.898) |

Note. ICC=intraclass correlation coefficient, ROI=region of interests, CI=confidence interval, SNR=signal-to-noise ratio, CNR=contrast-to-noise ratio, HDC=high dielectric constant

Table S2 Reported Overall Image Quality, Dielectric artifact and Diagnostic Confidence assessing images with and without HDC pad

|  |  | Reader 1 | | | | Reader 2 | | | | Reader 3 | | | | Overall | | | |
| --- | --- | --- | --- | --- | --- | --- | --- | --- | --- | --- | --- | --- | --- | --- | --- | --- | --- |
| Parameter |  | without HDC pad | | with HDC pad | | without HDC pad | | with HDC pad | | without HDC pad | | with HDC pad | | without HDC pad | | with HDC pad | |
| bSSFP group |  | n | % | n | % | n | % | n | % | n | % | n | % | n | % | n | % |
| Overall image Quality | 1 | 7 | 5.5 | 0 | 0.0 | 4 | 3.1 | 0 | 0.0 | 7 | 5.5 | 0 | 0.0 | 18 | 4.7 | 0 | 0.0 |
| Overall image Quality | 2 | 79 | 61.7 | 19 | 14.8 | 75 | 58.6 | 17 | 13.3 | 72 | 56.3 | 12 | 9.4 | 226 | 58.9 | 48 | 12.5 |
| Overall image Quality | 3 | 42 | 32.8 | 94 | 73.4 | 48 | 37.5 | 65 | 50.8 | 45 | 35.2 | 78 | 60.9 | 135 | 35.2 | 237 | 61.7 |
| Overall image Quality | 4 | 0 | 0.0 | 15 | 11.7 | 1 | 0.8 | 46 | 35.9 | 4 | 3.1 | 38 | 29.7 | 5 | 1.3 | 99 | 25.8 |
| Dielectric Artifact | 1 | 5 | 3.9 | 0 | 0.0 | 5 | 3.9 | 0 | 0.0 | 5 | 3.9 | 0 | 0.0 | 15 | 3.9 | 0 | 0.0 |
| Dielectric Artifact | 2 | 65 | 50.8 | 3 | 2.3 | 68 | 53.1 | 5 | 3.9 | 71 | 55.5 | 3 | 2.3 | 204 | 53.1 | 11 | 2.9 |
| Dielectric Artifact | 3 | 52 | 40.6 | 66 | 51.6 | 49 | 38.3 | 66 | 51.6 | 46 | 35.9 | 75 | 58.6 | 147 | 38.3 | 207 | 53.9 |
| Dielectric Artifact | 4 | 6 | 4.7 | 59 | 46.1 | 6 | 4.7 | 57 | 44.5 | 6 | 4.7 | 50 | 39.1 | 18 | 4.7 | 166 | 43.2 |
| Diagnostic Confidence | 1 | 2 | 1.6 | 0 | 0.0 | 3 | 2.3 | 1 | 0.8 | 4 | 3.1 | 0 | 0.0 | 9 | 2.3 | 1 | 0.3 |
| Diagnostic Confidence | 2 | 48 | 37.5 | 12 | 9.4 | 49 | 38.3 | 12 | 9.4 | 53 | 41.4 | 14 | 10.9 | 150 | 39.1 | 38 | 9.9 |
| Diagnostic Confidence | 3 | 58 | 45.3 | 53 | 41.4 | 54 | 42.2 | 57 | 44.5 | 50 | 39.1 | 61 | 47.7 | 162 | 42.2 | 171 | 44.5 |
| Diagnostic Confidence | 4 | 20 | 15.6 | 63 | 49.2 | 22 | 17.2 | 58 | 45.3 | 21 | 16.4 | 53 | 41.4 | 63 | 16.4 | 174 | 45.3 |
| SSFSE group |  |  |  |  |  |  |  |  |  |  |  |  |  |  |  |  |  |
| Overall image Quality | 1 | 5 | 12.5 | 0 | 0.0 | 4 | 10.0 | 0 | 0.0 | 2 | 5.0 | 0 | 0.0 | 11 | 9.2 | 0 | 0.0 |
| Overall image Quality | 2 | 6 | 15.0 | 0 | 0.0 | 12 | 30.0 | 3 | 7.5 | 12 | 30.0 | 3 | 7.5 | 30 | 25.0 | 6 | 5.0 |
| Overall image Quality | 3 | 21 | 52.5 | 13 | 32.5 | 17 | 42.5 | 17 | 42.5 | 21 | 52.5 | 19 | 47.5 | 59 | 49.2 | 49 | 40.8 |
| Overall image Quality | 4 | 8 | 20.0 | 27 | 67.5 | 7 | 17.5 | 20 | 50.0 | 5 | 12.5 | 18 | 45.0 | 20 | 16.7 | 65 | 54.2 |
| Dielectric Artifact | 1 | 6 | 15.0 | 0 | 0.0 | 5 | 12.5 | 0 | 0.0 | 6 | 15.0 | 0 | 0.0 | 17 | 14.2 | 0 | 0.0 |
| Dielectric Artifact | 2 | 9 | 22.5 | 0 | 0.0 | 11 | 27.5 | 2 | 5.0 | 8 | 20.0 | 3 | 7.5 | 28 | 23.3 | 5 | 4.2 |
| Dielectric Artifact | 3 | 15 | 37.5 | 11 | 27.5 | 16 | 40.0 | 14 | 35.0 | 14 | 35.0 | 17 | 42.5 | 45 | 37.5 | 42 | 35.0 |
| Dielectric Artifact | 4 | 10 | 25.0 | 29 | 72.5 | 8 | 20.0 | 24 | 60.0 | 12 | 30.0 | 20 | 50.0 | 30 | 25.0 | 73 | 60.8 |
| Diagnostic Confidence | 1 | 3 | 7.5 | 0 | 0.0 | 2 | 5.0 | 0 | 0.0 | 4 | 10.0 | 0 | 0.0 | 9 | 7.5 | 0 | 0.0 |
| Diagnostic Confidence | 2 | 7 | 17.5 | 0 | 0.0 | 9 | 22.5 | 1 | 2.5 | 5 | 12.5 | 3 | 7.5 | 21 | 17.5 | 4 | 3.3 |
| Diagnostic Confidence | 3 | 14 | 35.0 | 10 | 25.0 | 11 | 27.5 | 12 | 30.0 | 16 | 40.0 | 10 | 25.0 | 41 | 34.2 | 32 | 26.7 |
| Diagnostic Confidence | 4 | 16 | 40.0 | 30 | 75.0 | 18 | 45.0 | 27 | 67.5 | 15 | 37.5 | 27 | 67.5 | 49 | 40.8 | 84 | 70.0 |

Note. n=Number of observations. bSSFP=balanced steady state free precession. SSFSE=single-shot fast spin-echo.
